# Workplace resilience and compassionate care among Jordanian private sector nurses

**DOI:** 10.1186/s12912-024-02295-z

**Published:** 2024-09-11

**Authors:** Yousef Mohammad Nassar, Nidal Eshah, Hindya O. Al-Maqableh, Abdulqadir J. Nashwan, Ahmad Rayan, Mohammad J. Alhawajreh

**Affiliations:** 1 Faculty of Nursing, Zarqa University, Zarqa, Jordan; 2 Health Services Administration, Faculty of Medicine, Yarmouk University, Irbid, Jordan; 3 Nursing & Midwifery Research Department, Hamad Medical Corporation, Doha, Qatar

**Keywords:** Compassionate care, Workplace resilience, Nurse manager, Nurses, Jordan

## Abstract

**Background:**

Compassionate care is a hallmark of the nursing profession. Yet, nursing is beset by perennial problems, not the least of which is nursing shortage and increased workload. As such, resilience becomes a critical ingredient that nurses must possess to overcome such challenges. However, there needs to be more evidence of the relationship between compassionate care and resilience within the Jordanian nursing context.

**Aim:**

To explore the relationship between workplace resilience and compassionate care among Jordanian nurses working in the private sector.

**Methods:**

The study utilized a descriptive cross-sectional correlational design. Convenience sampling with inclusion-exclusion criteria was used to select participants from three private hospitals in Jordan. The Compassionate Care Questionnaire was used to measure levels of compassionate care, and the Resilience at Work Scale was used to measure workplace resilience. Ethical approval was obtained before data collection.

**Results:**

A total of 161 nurses participated in the study. Participants had high levels of compassionate care and workplace resilience. Male nurses and nurses with lower workloads had significantly higher levels of compassionate care. Likewise, older nurses, nurses with postgraduate degrees, and nurses with experience of less than 5 years in the current area had significantly higher levels of workplace resilience. Compassionate care had a mordantly solid and significant positive relationship with workplace resilience and all its seven dimensions (living authentically, finding one’s calling, maintaining perspective, managing stress, interacting cooperatively, staying healthy, and building networks.

**Conclusion:**

Developing workplace resilience can support nurses in implementing compassionate care. Nurse Managers and hospital administrators must consider the effects of compassionate care and workplace resilience on nurses and patients. Future research can include a longitudinal exploration of compassionate care and workplace resilience and an investigation of the levels of these variables outside a hospital setting.

## Introduction

### Background

>Nursing is a demanding and stressful profession. Nurses are exposed to a heavy workload when there is an acute shortage of staff or have to handle two assignments at the same time, and it becomes more challenging to fulfill their roles and responsibilities unless they can demonstrate an adequate ability to cope with the stress and pressure that associated with clinical workload [1]. Resilience is critical in these scenarios, emerging as a vital component of the nursing profession. A recent integrative review explored the concept of resilience among nurses; the result of the review showed that the ability to adapt to the healthcare system’s stresses positively, a psychological capacity to meet work challenges, as a personal attribute, a protective characteristic and a method of cognitive reframing to maintain positive workplace relationships and balance [2].on one hand, The resilience positively correlates with hardiness, self-esteem, self-efficacy, life and job satisfaction, on other hand, it negatively correlates with depression, anxiety, stress, and burnout [2]. Another study found that higher educational attainment, lower levels of anxiety, and effective coping strategies before shifts are significant predictors of resilience among nurses [3].

Research explored the critical role of a resilient nursing environment and the role of nursing management in shaping a supportive work environment that can significantly influence nurse’s ability to perform their duties [4]. Moreover, a study for critical care nurses in Malaysia demonstrated that the quality of the nursing work environment significantly predicted nurse resilience and, in turn, their intention to leave [5].

Caring is central to nursing, distinguishing it from other healthcare professions. Caring involves prioritizing the best interest and is characterized by helpful, empathy, understanding, active listening, and desire to do well to others, collectively contributing to compassionate care [6, 7]. Compassionate care is understood as arising from the true concern to elevate the suffering of others; the research has further defined compassionate care within clinical settings, described as a combination of empathy, alleviating suffering, addressing individual patient needs, and effective communication – all influenced by nursing leadership and workplace cultures [8, 9].

This synthesis aims to bridge the research gap by examining the relationship between resilience and compassionate care whining the nursing profession, especially in the challenging work environment.

### Problem statement

A descriptive cross-sectional study examined the relationship between 321 Saudi critical care nurses’ personal characteristics, coping methods, and resilience. The result showed that the length of work shift, educational level, and nationality were significantly connected with resilience; the investigators only linked resilience with compassion fatigue and despondency when caring for critically sick patients [10]. A recent study found that structured personal reflective debriefing improved emergency nurses’ resilience but not compassionate care [11]. A study suggested developing emotional intelligence and mindfulness to build resilience in nurses and healthcare teams, but no direct link was found to compassionate care [12]. Another research examined how resilience affects nurses’ stress and quality of life in Jordan; the result showed that nurses who had a higher level of resilience had lower levels of stress, which enhanced their quality of life but not compassion towards patients [13]. Also, is study found that reduced amount of sleep and leisure increased burnout risks and decreased compassion, but no evidence that an increased level of adaptation to stress improved compassion satisfaction [14]. In a study of 228 Jordanian nurses, the investigators examined nurses’ coping strategies and compassion fatigue; the result found that good coping skills reduce stress and improve emotional stability and compassion. Although these studies suggest a link between resilience and compassionate care, the investigator needs to provide empirical data to fill the knowledge gap.

### Significance of the study

The study enhances nurses’ self-awareness and quality of care by promoting compassionate care, also, it can help nurses to understand the value of resilience to be still able to provide safe and quality patient care despite challenges in their workplaces [2]. It analyzes workplace needs to improve management and organizational impact [8]. Insights aid nurse educators in creating teaching plans that promote compassionate care and resilience [16]. Policymakers can use the study to support healthcare policies that foster compassionate care and resilience, ensuring better patient outcomes [23]. Lastly, the study benefits patients and their families by receiving care from nurses who consider compassionate care and resilience critical to their profession.

### Purpose of the study

The study aims to assess the relationship between workplace resilience and compassionate care among Jordanian nurses working in the private sector.

### Research objectives

The study aims to assess workplace resiliency and compassionate care among Jordanian private-sector nurses, identify associated sociodemographic and professional characteristics, and determine if a significant relationship exists.

### Research questions

The research questions were:

What is the level of compassionate care among nurses working in the private sector in Jordan?

What is the level of workplace resilience among nurses working in the privet sector in Jordan?

Are there differences or relationships in the level of compassionate care and workplace resilience based on sociodemographic characteristics?

Is there a relationship between workplace resilience and compassionate care among Jordanian nurses working in the private sector?

### Conceptual definitions

This study defines key variables as resilience, which refers to an individual’s ability to manage work stress, rebound from setbacks, and prepare for future challenges [16], and compassionate care, which is an expression of shared professional morality expected by nurses, patients, and society [17].

### Operational definitions

The following are how key variables are operationally defined in this study:

1. Resilience – is defined as the ability of a nurse to cope with and overcome stress, difficulty, and distress associated with daily clinical work and is measured using the Resilience at Work (RAW) Scale [18].
2. Compassionate care – is defined as the ability of the nurse to provide care that is sensitive and responsive to the needs of the patient and family, and that arises from the nurse’s genuine desire to help and is measured using the Schwartz Centre Compassionate Care (SCCC) Scale [19].

## Literature review

### Search strategy

The study reviewed the current understanding of workplace resilience and compassionate care. It used electronic databases like CINAHL, Medline, Ovid, and Google Scholar. The studies were screened for eligibility based on inclusion-exclusion criteria, including original research, quantitative, qualitative, or mixed-method designs, and a focus on workplace resilience and compassionate care. Limiters included publication date, English, peer-reviewed journal, and full-text availability. Quality assessments were conducted using critical appraisal checklists.

### Workplace resilience

Nurse resilience is crucial for coping with the demands of their profession. An integrative review of 27 studies identified three main themes: personal characteristics enhancing resilience, workplace resilience characterized by overcoming adversity adaptive strategies, and a supportive environment like peer support and job satisfaction. A recent study [20] highlighted the importance of social support, work-life balance, and self-care in resilience-building. New research [21] investigated personal and work-related factors associated with nurse resilience, revealing that measurements vary significantly, affecting consistency in identifying influential factors.

Organizational culture is crucial in nurse job satisfaction, well-being, and resilience. Effective management of workplace stressors and supportive leadership can enhance resilience. A study [22] found that individual attributes like mindfulness and organizational attributes like supportive leadership are essential for improving workplace resilience. Several studies [15, 23] found that leadership strategies like fostering social connections and self-care significantly boost staff resilience. Various studies [24, 25] explored strategies for nurses to build resilience in workplace adversity.

 [26]. Other studies suggest that nurse resilience is influenced by personal characteristics and workplace demands, especially during crises like the COVID-10 pandemic. Different studies [12, 27] highlight the need for strategies to enhance resilience among Jordanian nurses, highlighting the potential benefits of improving well-being and care quality. These studies advocate for comprehensive approaches to building resilience and strengthening nurses’ professional skills.

### Compassionate care

Compassion in nursing care is crucial for improving healthcare delivery systems. A study [28] defined compassionate care as ethical, professional, practical communication, human, spiritual, religious, and patient involvement. They emphasized the impact of workload, role modeling, value systems, and organizational culture on compassionate care. Several investigations [29, 30] further refined the understanding of compassionate care, describing it as an empathic process where nurses actively communicate and address patient concerns. The current study [31] highlighted the impact of internal markets and managerialism on compassionate care criteria and nursing quality and safety. A study [17] found workplace culture and peer support as factors affecting compassionate care intensive care, highlighting trust, collaboration, and empathy in Norwegian palliative care compassion. Various studies [9, 32] identified barriers to compassionate care, such as hospital environments, sociocultural climates, and staff nurses’ attitudes.

## Method

### Research design

The study used a descriptive cross-sectional correlational research design to examine the relationship between workplace resiliency and compassionate care among private clinical nurses in Jordan. It focused on sociodemographic characteristics and measured levels during a specific period.

### Research setting

The study was carried out in three private hospitals in Jordan following the result of the literature review suggesting the gap in evidence about workplace resilience and compassionate care among nurses working in private sector hospitals and these hospitals located in the Jordanian capital Amman. The study was carried out in the following hospitals, namely, Israa Hospital, with (60) registered nurses; Islamic Hospital, with (131) registered nurses; and Istiqlal Hospital, with (80) registered nurses.

### Sampling frame

The study used a convenience sampling design with inclusion-exclusion criteria to recruit participants. The inclusion criteria required registered nurses employed by the participating hospital research site and at least 6 months of experience. On the other hand, Exclusion criteria excluded nurses with disability, diploma degrees, and working in operating rooms, outpatient departments, or managerial positions with minimal patient contact. G* Power version 3.1.9.7 was used to calculate the sample size. The target minimum sample size was 161 nurses from the total accessible population of 271 registered nurses. They were assuming a moderate effect size of 0.5 due to the lack of previous studies on workplace resilience and compassionate care among nurses in Jordan.

### Data collection

The researcher collaborated with hospital heads to gather data from eligible nurses. They obtained a list of nurses and invited them to participate. After informed consent, they completed survey questionnaires via WhatsApp groups. The questionnaires were brief and took 6–10 min to complete. Data was stored in an encrypted computer and destroyed based on Jordan’s data privacy and protection laws.

### Pilot test

A pilot test was conducted to assess the feasibility of the research design and the usability of online survey questionnaires. The test had a 10% sample size, which allowed for troubleshooting issues and adhering to ethical principles. The sample was from the accessible population and met inclusion and exclusion criteria. The pilot testing outcome introduced a road map for planning our study as the education level significantly impacted the level of resiliency, so all participants should have a bachelor’s degree as a minimum.

### Measurements

#### Participant information sheet

The researcher created a participant information sheet to gather sociodemographic data of participants, ensuring anonymity and confidentiality. The sheet included details about the participant’s age, gender, education, area of practice, years of experience as a registered nurse year of experience in current department, and workload.

#### Compassionate care questionnaire

The study utilized the Schwartz Centre Compassionate Care Scale (SCCCS) [19]. A study was also conducted to measure compassionate healthcare providers using SCCCS. The result was excellent psychometric properties by Classical Test Theory and Rasch measurement theory, and it introduced more understanding and discussion for compassionate healthcare providers. A questionnaire designed to measure the level of compassionate care provided by healthcare professionals [33]. The tool has 12 items, each scored on a 10-point Likert scale, with a minimum score of 12 and a maximum score of 120, indicating a higher level of compassionate care. The questionnaire is reliable and valid, with a Cronbach alpha of 0.98 and a good model fit (CFI = 0.92) through exploratory and confirmatory factor analysis.

#### Resilience at work (RAW) scale

The Resilience at Work (RAW) Scale [18], is a tool used to measure personal resilience in the workplace. Moreover, many studies are using this scale to assess resilience at work. For instance, a study was conducted to explore the assessment of resilience at work among 1st-line nurse managers using the RAW scale, and the result showed first-line managers have a high level of resiliency [34]. The questionnaire consists of 25 items covering seven components: living authentically, finding one’s calling, maintaining perspective, managing stress, interacting cooperatively, staying healthy, and building networks. The overall score ranges from 0 to 150, with higher scores indicating higher resilience levels [18]. Resilience at work scale has acceptable levels of reliability and validity. Cronbach alpha for the whole questionnaire was 0.84, and convergent validity was significant (*p* < 0.001); many studies found similar robust psychometric properties [18, 35].

### Ethical considerations

The study was conducted with the approval of the Institutional Review Board (IRB) of Zarqa University and selected hospital administrators to ensure ethical standards were met in recruitment, data collection, data protection, and usage. Participants’ anonymity was maintained, and informed consent was obtained from nurses. The study aimed to understand the relationship between workplace resiliency and compassionate care, helping nurse managers develop strategies for improving these aspects. Nurses were assured that participating would not negatively impact their relationships with the hospital or employers. The study involved no experimentation or intervention. To ensure data confidentially, access to all collected data was limited to the author. The data is stored in the encrypted password-protected password-protected computer and will be destroyed at the end of publication based on prevailing data privacy and data protection laws in Jordan.

### Data analysis

The study analyzed electronic data of nurses working in private clinical settings in Jordan using SPSS version 25. Data was cleaned and examined to remove duplicates, errors, and missing items. Descriptive statistics were used to measure sociodemographic characteristics, compassionate care, and workplace resiliency. Inferential statistics were used to test the correlation between workplace resilience and compassionate care. Independent t-tests were used to measure differences in compassionate care and workplace resiliency based on gender, educational attainment, area of practice, length of years as a registered nurse, and length of years employed in the current work area. Pearson’s r was used to test relationships between compassionate care and workplace resiliency. also, it is used to test relationships between compassionate care and workplace resiliency based on age and amount of workload. One-way ANOVA analysis was used to measure differences in levels of compassionate care and workplace resiliency based on the work area.

## Results

### Sample characteristics

The study participants included 161 nurses. The participants had a mean age of 31 years (SD ± 5.1 years); furthermore, the majority of them were male (*n* = 113, 70.2%), and the rest were female (*n* = 48, 29.8%). Also, most of them had undergraduate degrees (*n* = 142, 88.2%), and the rest were postgraduate (*n* = 19, 11.8%), and the majority of them worked in specific units (intensive care, cardiology, and neonatal intensive care). Moreover, most registered nurses have less than 5 years of experience (*n* = 109, 67.7%). When we questioned their years of experience in the current department, most participants have worked for more than 5 years(*n* = 97, 60.2%). Three hospitals recruited participants: Islamic Hospital (*n* = 64, 39.8%), Al-Esraa Hospital (*n* = 63, 39.1%), and Istiqlal Hospital (*n* = 34, 21.1%). The workload averaged 7 patients each shift (SD ± 6), as shown in (Table 1).

**Table 1:** Sociodemographic characteristics and work history of the sample (*n* = 161)

Variable	Frequency (*n*)	Percentage (%)
Gender		
Male	113	70.2
Female	48	29.8
Educational Attainment		
Bachelor	142	88.2
Master’s or PhD	19	11.8
Hospital		
Islamic Hospital	64	39.8
Istiqlal Hospital	34	21.1
Al-Esraa Hospital	63	39.1
Area of practice		
Wards	70	43.5
Special Units (ICU, CCU, NICU)	91	56.5
Experience as RN		
≤ 5 years	109	67.7
> 5 years	52	32.3
Years Working in the Current Department		
≤ 5 years	64	39.8
> 5 years	97	60.2

### Compassionate care

Research question: What is the level of compassionate care among nurses working in the private sector in Jordan?

Compassionate care was measured using the Schwartz Centre Compassionate Care Scale, which produces a range of scores from a minimum of 12 to a maximum of 120 [19]. The mean score of compassionate care among the study participants was 93.5 (*SD* ± 18.0). The minimum score was 40, and the maximum score was 120, with a range of 80.

### Workplace resilience

Research question: What is the level of workplace resilience among nurses working in the privet sector in Jordan?

Workplace resilience was measured using the Resilience at Work Scale, which produces a range of scores from a minimum of 0 to a maximum of 150 [18]. The mean RAW score among the study participants was 99.4 (*SD* ± 21.8), the minimum resilience at work score recorded as 16.33, and the maximum score recorded as 141 among the study, with a range of 124.7 as shown in (Table 2).

**Table 2:** Mean score for each of the subdomains of workplace resilience

Subdomains of Workplace Resilience	Mean	SD ±
Maintaining perspective	26.1	5.7
Finding One’s Calling	28.3	9.9
Managing stress	29.3	8.3
Living authentically	29.9	9.7
Staying healthy	32.7	10
Building network	32.8	10
Interacting cooperatively	33.8	9.6
**Overall score**	**99.4**	**21.8**

### Workplace resilience and compassionate care based on sample characteristics

Research question: Are there differences or relationships in the level of compassionate care and workplace resilience based on sociodemographic characteristics?

Person R was used to investigate the relationship between workplace resilience and age. The results show a positive and weak relationship between them (*r* = 0.16, *p* < 0.05). Also, person R was used to examine the relationship between compassionate care and workload the results show a significant but weak negative relationship (*r*= -0.18, *p* < 0.05). Thus, nurses caring for more patients have lower of compassionate care. The independent sample t-test was used to examine workplace resilience with educational level and experience in the current department; the results show that postgraduate nurses have higher resilience than undergraduates ( *p* < 0.05), as shown in (Table 3), as well as nurses with experience less than 5 years in current area have higher resilience than more than 5 years experience ( *p* < 0.05) as shown in (Table 3). Furthermore, the independent sample t-test was used to examine the level of compassionate care with gender; the result found that male nurses had more compassionate care than females ( *p* < 0.05), as shown in (Table 3).

**Table 3:** Differences in workplace resilience and compassionate care based on participant sociodemographic characteristics and work history

Variable	Category	CC	RAW
		Mean (SD±)	Mean (SD±)
Gender	Male	94.2 (16.1)	99.2 (21.7)
	Female	91.8 (21.9)	99.7 (22.2)
	T	0.78	-0.12
	P-value	0.01*	0.81
Educational Attainment	Bachelor	92.9 (18.4)	99.0 (22.8)
	Master, PhD	98.1 (13.7)	102.0 (12.4)
	T	-1.18	-0.57
	P-value	0.15	0.03*
Area of Work	Wards	91.9 (17.7)	97.5 (21.1)
	Special Unit	94.7 (18.2)	100.8 (22.3)
	T	-1.00	-0.96
	P-value	0.81	0.81
Years of Experience as an RN	≤ 5 years	93.1 (18.0)	101.1 (20.2)
	> 5 years	94.3 (18.1)	95.6 (24.6)
	T	-1.50	0.42
	P-value	0.06	0.92
Years of Experience in the Current Department	≤ 5 years	93.6 (18.3)	103.3 (16.3)
	> 5 years	93.4 (17.8)	96.7 (24.5)
	T	-0.05	-1.90
	P-value	0.47	0.02*

Legend: CC – compassionate care, RAW – workplace resilience, * - significant at *p* < 0.05

### Association between compassionate care and workplace resilience

Research question: Is there a relationship between workplace resilience and compassionate care among Jordanian nurses working in the private sector?

Pearson’s *correlation coefficient* was used to test if significant relationships existed between compassionate care and workplace resilience. The results showed that compassionate care had a significant and moderately strong positive relationship with workplace resilience (*r* = 0.46, *p* < 0.01). A positive relationship was also found between compassionate care and the seven sub-dimensions of workplace resilience, namely Living Authentically (*r* = 0.40, *p* < 0.01), Finding One’s Calling (*r* = 0.40, *p* < 0.01), Maintaining Perspective (*r* = 0.24, *p* < 0.01), Managing Stress (*r* = 0.44, *p* < 0.01), Interacting Cooperatively (*r* = 0.29, *p* < 0.01), Staying Healthy (*r* = 0.36, *p* < 0.01), and Building Networks (*r* = 0.37, *p* < 0.01).

## Discussion

### Compassionate care

The study results showed that participants had high scores on compassionate care. However, it is challenging to analyze the high levels of compassionate care obtained in this study compared to other Jordanian nurse cohorts because this concept was seldom measured and expressed as a quantitative variable within the Jordanian healthcare context. A previous exploration of compassionate care was performed on a sample of nurses working in four Jordanian hospitals. Even in that study, the authors did not measure compassionate care [14]. Other international studies did not measure compassionate care levels but explored its qualitative dimension. For instance, studies carried out in the UK [9, 31], Australia [17], and Iran [1, 29, 32] explored the qualitative aspect of compassionate care. Nevertheless, the high scores on compassionate care among the participants suggested the need to continue enriching and nurturing this desirable characteristic among Jordanian nurses while also recognizing the need to perform other studies to assess the Jordanian nurses’ compassionate carers and what their experiences are when trying to be compassionate towards patients and their families. When nurses are compassionate, they become more acutely aware of the needs of their clients, increasingly responsive to meet demands and requests, understand the unique perspectives and preferences grounded on culture, values, and beliefs, advocate and promote the health, safety, well-being and best interests of their patients; and create environments that are supportive and conducive for healing and rehabilitation.

### Workplace resilience

The current study results show a high level of resilience based on resilience at the work scale among Jordanian nurses working in the private sector. The findings showed that Jordanian nurse participants had higher resilience levels than Greek [3] and American [34] samples. However, another study showed lower levels of workplace resilience in their sample of Jordanian nurses [12]. The controversy between the current research results and that suggests heterogeneity in the values of workplace resilience, so the level of workplace resilience varies across settings and the prevailing cultures, conditions, and circumstances surrounding healthcare organizations [36]. Moreover, the inconsistency of results suggests the need for nurse managers and hospital administrators to look into ways to improve workplace resilience among staff nurses and maintain standards that ensure the health and well-being of staff, patients, and other service users within the healthcare organization. In terms of research, measuring workplace resilience against work environment characteristics might help explain the variances in measured levels of workplace resilience among individual staff nurses.

Likewise, when nurses have high levels of workplace resilience, they have the capacity and flexibility to meet the ever-growing demands of the profession without succumbing to the adverse effects of stress, depression, and anxiety. Compassionate and resilient nurses are uniquely positioned to provide excellent patient care despite the pressures of the healthcare system.

### Sociodemographic characteristics and compassionate care

The results showed that male nurses and nurses with lower workloads demonstrated higher levels of compassionate care. Interestingly, male nurses had higher scores on compassionate care than their female counterparts, considering that some previous studies have noted the other way around or at least an equivalence or similarity [38, 39]. More so considering societal expectations that males are more reserved in expressing their emotions and that patient perspectives point toward expecting more caring behaviors from females than male nurses [38, 39]. for instance, a study found significantly higher patient expectations of compassionate nursing care from female than male nurses [38]. On the other hand, a recent study pointed out that while male nurses are respectful, good listeners, unbiased, and supportive, they can foster such behaviors further to improve their relationships with patients [39]. Future research can look into the reasons behind this finding, especially as there is not enough data from the study to hypothesize why this might be so, although this might be explained by the higher proportion of male nurses in the sample compared to female nurses.

On the other hand, nurses with higher workloads were seen to have significantly lower scores on compassionate care. This is because a higher number of patients being cared for would mean more tasks that need to be completed, and the sheer volume would make it difficult for nurses to perform other activities that reduce compassionate behavior [40, 41]. For instance, a meta-analysis study found higher nurse-to-patient ratios were associated with higher levels of burnout, job dissatisfaction, and intent to leave, which, in turn, negatively affects the ability of nurses to extend compassionate care to patients [41]. So, wards must maintain an appropriate nurse-patient ratio and skill mix to ensure that nurses can still demonstrate kind and caring behaviors despite the work that needs to be completed.

### Sociodemographic characteristics and workplace resilience

The results showed that older nurses and nurses with postgraduate degrees had significantly higher levels of workplace resilience, suggesting that nurses with more maturity and educational background manifested better abilities to cope with professional demands and pressures. The experience brought about by age and education equips nurses with the knowledge and skills to confront specific patient demands, regulatory requirements, ward activities, and other goals and objectives that need to be met [44], highlighting that nurse maturity significantly contributes to workplace resilience, as experience could help nurses plan, prioritize, and address their tasks and workloads. However, two integrative reviews noted that individual attributes such as sociodemographic characteristics of age, gender, and years of experience were not significantly associated with workplace resilience [22, 36]. Nevertheless, all authors agree that there might be a benefit in designing and implementing interventions that will support junior nurses and provide educational training to develop better competencies that will allow nurses to cope effectively with facing workplace challenges. Moreover, there is a need to conduct workshops for nurse managers that focus on the importance of a positive workplace environment to enhance the familiarization between nurses and staff, promoting workplace resilience, which will be reflected positively in compassionate care. Lastly, there is a need to provide the necessary resources that help create a flexible work environment.

However, it is worth noting that high levels of workplace resilience were not detected based on years of experience as a registered nurse; instead, nurses with less than 5 years of experience in the current hospital department had higher levels of workplace resilience than those who had more than 5 years of experience. Going back to the link between professional experience and workplace resilience suggests that more senior, experienced nurses would have higher levels of resilience owing to their maturity, level of competencies, educational preparation, and familiarization. This result is consistent with those who noted a positive work environment and favorable work characteristics with good leadership support enhanced individual levels of resilience [45]. The findings need to have adequate data to make inferences about why this is so. therefore, future research can elucidate the reasons behind this finding.

### Compassionate care and workplace resilience

The study found a significant positive relationship between compassionate care and workplace resilience in the Jordanian context, highlighting the importance of nurse managers and hospital administrators working towards improving these characteristics to maintain an adaptive, responsive, and caring nursing workforce. Previous studies have indirectly explored this relationship [12, 32].

### Strengths and limitations

The study explores the relationship between compassionate care and workplace resilience of Jordanian nurses in various hospital settings. It highlights how compassion can help nurses cope with workplace pressures without compromising patient care quality. The study uses reliable instruments, adequate sample size, and clear inclusion-exclusion criteria.

On the other hand, the study has some limitations. First, the study is cross-sectional, which means there were no attempts to measure the levels of compassionate care and workplace resilience over time. While it may appear that compassionate care is a static variable, workplace resilience might possess more dynamism, given that professional pressures and work demands are constantly changing depending on the prevailing status of the healthcare delivery system. Second, the study did not attempt to investigate factors that might predict the levels of compassionate care and workplace resilience, and the exploration of factors associated with the two primary variables was limited to sociodemographic characteristics. The sample was mostly made up of nurses working in private hospital settings. As such, the study results have limited generalizability to nurses in other healthcare settings. The study utilized convenient sampling, meaning there might be some risk of selection bias. Compared to randomization, the non-probability sampling only allowed the participation of present or available nurses at the time of data collection. Not all nurses had an equal chance to be selected.

### Conclusions

The study examined the correlation between compassionate care and workplace resilience among nurses in Jordanian hospitals. Results showed that male nurses and those with lower workloads had higher compassionate care scores, while older, postgraduate-educated nurses had higher workplace resilience scores. Moreover, nurses with high scores on compassionate care had significantly higher scores on workplace resilience. Future research on compassionate care and workplace resilience needs to focus on knowing what intervention can promote the level of workplace resilience and compassionate care. Also, investigating the levels of these variables outside of a hospital setting and exploring organizational factors for these variables are recommended areas for future research. Stakeholders and hospital administrators must consider the effects of compassionate care and workplace resilience on both nurses and patients.

### Implications

#### Implications for practice

Resilience in the workplace involves effective coping mechanisms, communication, leadership, prioritization, time management, and teamwork skills. Nurses must provide compassionate, empathic, and understanding care to patients and their families. Nurse Managers should review staffing and patient load regularly, ensuring target nurse-patient ratios. Additionally, they should focus on retaining and sustaining current nursing workforces and intensifying recruitment drives to fill vacancies.

#### Implications for policy

Nurses need policies to effectively use coping mechanisms, access social networks for support, and receive counseling and recreation services to cope with daily practice demands and pressures. Nurse managers should also offer these services.

#### Implications for research

The study identified sociodemographic variables affecting compassionate care and workplace resilience among Jordanian nurses but lacked organizational characteristics data, so we need further research to investigate organizational factors.

#### Implications for education

Nurses with higher educational attainment show higher workplace resilience. Nurse educators should develop programs and workshops to improve resilience and compassionate care. These programs help nurses adapt to work stressors and provide patient-oriented care.

### Recommendations

Nurse researchers should conduct studies on individual and organizational factors affecting compassionate care and workplace resilience, as well as the effects of sociodemographic and work history. They should also explore effective interventions and their impact on patient outcomes like hospital stay, mortality, satisfaction, and quality of life. Future research should also examine the relationship between compassionate care and nurse outcomes.

## Data Availability

The data supporting this study’s findings are available from the corresponding author upon reasonable request.
