# Correction: Workplace resilience and compassionate care among Jordanian private sector nurses

**DOI:** 10.1186/s12912-024-02361-6

**Published:** 2024-09-27

**Authors:** Yousef Mohammad Nassar, Nidal Eshah, Hindya O. Al-Maqableh, Abdulqadir J. Nashwan, Ahmad Rayan, Mohammad J. Alhawajreh

**Affiliations:** 1 Faculty of Nursing, Zarqa University, Zarqa, Jordan; 2 Health Services Administration, Faculty of Medicine, Yarmouk University, Irbid, Jordan; 3 Nursing & Midwifery Research Department, Hamad Medical Corporation, Doha, Qatar

**BMC Nursing (2024) 23:634**

10.1186/s12912-024-02295-z

The original article displayed a small typo in Ahmad Rayan’s name which has since been amended.

